# Psychiatric advance directives for people living with schizophrenia, bipolar I disorders, or schizoaffective disorders: Study protocol for a randomized controlled trial – DAiP study

**DOI:** 10.1186/s12888-019-2416-9

**Published:** 2019-12-27

**Authors:** Aurélie Tinland, Léa Leclerc, Sandrine Loubière, Frederic Mougeot, Tim Greacen, Magali Pontier, Nicolas Franck, Christophe Lançon, Mohamed Boucekine, Pascal Auquier

**Affiliations:** 1 School of medicine – La Timone Medical Campus, EA 3279: CEReSS – Health Service Research and Quality of Life Center, Aix-Marseille University, 27 Boulevard Jean Moulin, F-13005 Marseille Cedex 5, France; 2 Department of Psychiatry, Sainte-Marguerite University Hospital, F-13009 Marseille, France; 3 Department of Clinical Research and Innovation, Support Unit for clinical research and economic evaluation, Assistance Publique – Hôpitaux de Marseille, F-13385 Marseille, France; 4 Centre Max Weber, UMR 5283 – Centre hospitalier Le Vinatier, Lyon, France; 5 Mental Health and Social Sciences Research Unit, Paris Psychiatry and Neurosciences University Hospital Group, Paris, France; 6 Resource center of psychosocial rehabilitation, Centre hospitalier Le Vinatier, UMR 5229, CNRS & Université Claude Bernard Lyon 1, Université de Lyon, Lyon, France

**Keywords:** Facilitated psychiatric advances directives, Advance decision making, Supported decision making, Peer support workers, Compulsory admission

## Abstract

**Background:**

Compulsory admission to psychiatric hospital is rising despite serious ethical concerns. Among measures to reduce compulsory admissions, Psychiatric Advance Directives (PAD) are the most promising, with intensive PAD (i.e. facilitated and shared) being the most effective. The aim of the study is to experiment Psychiatric Advance Directives in France.

**Methods:**

A multicentre randomized controlled trial and qualitative approach conducted from January 2019 to January 2021 with intent-to-treat analysis.

**Setting:**

Seven hospitals in three French cities: Lyon, Marseille, and Paris. Research assistants meet each participant at baseline, 6 months and 12 months after inclusion for face-to-face interviews.

**Participants:**

400 persons with a DSM-5 diagnosis of bipolar I disorder (BP1), schizophrenia (SCZ), or schizoaffective disorders (SCZaff), compulsorily admitted to hospital within the last 12 months, with capacity to consent (MacCAT-CR), over 18 years old, and able to understand French.

**Interventions:**

The experimental group (PAD) (expected *n* = 200) is invited to fill in a document describing their crisis plan and their wishes in case of loss of mental capacity. Participants meet a facilitator, who is a peer support worker specially trained to help them. They are invited to nominate a healthcare agent, and to share the document with them, as well as with their psychiatrist. The Usual Care (UC) group (expected *n* = 200) receives routine care.

**Main outcomes and measures:**

The primary outcome is the rate of compulsory admissions to hospital during the 12-month follow-up. Secondary outcomes include quality of life (S-QoL18), satisfaction (CSQ8), therapeutic alliance (4-PAS), mental health symptoms (MCSI), awareness of disorders (SUMD), severity of disease (ICG), empowerment (ES), recovery (RAS), and overall costs.

**Discussion:**

Implication of peer support workers in PAD, potential barriers of supported-decision making, methodological issues of evaluating complex interventions, evidence-based policy making, and the importance of qualitative evaluation in the context of constraint are discussed.

**Trial registration:**

ClinicalTrials.gov identifier: NCT03630822. Registered 14th August 2018.

## Background

Persons with mental illness can experience fluctuations in their state of mental health, with their mental capacity being more or less altered [1]. In cases where the person becomes incapable of making decisions and needs care, the use of compulsory care is legally possible and organized with the objective to prevent self-harm and harm to others [2]. When compulsory treatment is used, the principle of benevolence prevails over the principle of autonomy in the ethical balance of the clinician [3]. However, an increasing number of studies show the importance of autonomy and self-determination in the recovery from severe mental disorders [4–8], supported by the claims of activists and legal texts of human rights bodies [9]. Furthermore, a growing number of studies show the harmful impact of compulsory treatment on the person [10–17]. These two bodies of evidence complicate the assessment of the weight of each ethical argument and bring many dilemmas [18, 19], but do not systematically lead to a decrease in this type of measure. Indeed, a recent review founded an increase in involuntary admission in 11 of 18 countries surveyed, including France [20]. The published rates vary with a ratio of 1 to 19.5 between comparable Western European countries (i.e. Italy and Austria) [20], and between departments within the same country (e. g. in France with a ratio of 1 to 6 [21]. Beyond hospitalization, very wide disparities are observed throughout the field of research on coercion, that depend on culture, mental health legislation, social context (socioeconomic characteristics, urbanisation) or service configuration [20, 22].

These disparities do not inhibit action, and the meta-analysis of de Jong and coll [23]. identified several robust studies on interventions to reduce compulsory hospital admission for persons with severe mental illness, with four main relevant evaluated approaches: advance statements, community treatment orders, compliance enhancement, and integrated treatment. Among these interventions, advance statements were the most promising, showing a statistically significant and clinically relevant reduction in compulsory admissions for adults in psychiatric hospital [23].

In literature, terms “advance statements”; “crisis planning”, “treatment preferences” and “advance directives” are found, with advance directives being the widest spread [20]. Psychiatric advance directives (PADs) are written documents that allow adults with decision-making ability to declare their care preferences in advance, in order for them to be applied in the event of an impairment of this ability [24]. Specific forms of PADs are described such as crisis cards, treatment plans, Ulysses Directives, Joint Crisis Plan (JCP), facilitated psychiatric advance directives, care plan, advance care plan, advance decision-making, and the Wellness Recovery Action Plan (WRAP) [25–31]. All these forms of PAD have treatment preferences in common but differ in legal framework, the presence or not of a facilitator, the sharing conditions, and their content, in particular their integration in a self-management plan, and the designation of a health care agent to act on behalf of the person should he or she be deemed mentally incapable in the future [32–36]. PADs are considered to be a complex intervention, with a) several interacting components including the document itself, support by the facilitator, professional and family context, and b) several relevant levels of implementation including completion of the document, content of the document, access to directives by stakeholders and compliance with directives [37].

Considering current literature on the effects of PADs in reducing compulsory admissions as primary outcome, five RCTs of high methodological quality were conducted [38–42]. A recent meta-analysis including these five articles showed a 25% reduction in compulsory admissions for people with PADs compared to usual care (risk ratio 0.75, 95% CI 0.61–0.93, *P* = 0.008) [33], with a greater effectiveness of intensive PADs (i.e. facilitated by an healthcare agent and shared with caregivers) among the different models used.

Beyond their effectiveness on constraint, PADs have shown an improvement of empowerment and self-determination, awareness, comprehension and appropriation of symptoms and partnership [25, 26, 28, 43–48].

All studies highlight the importance of one-to-one facilitation to improve drafting, understanding, and sharing of PADs [25, 28, 33].

In France, advance directives were created by the law of April 22, 2005 and mainly used in end-of-life healthcare [49]. PADs are only marginally used by a few pioneering teams. Specifically in Marseille, a group defending the rights of persons living with mental health problems has adopted an advance directive document used in a preliminary study and based on the JCP model. From there, the group has developed a peer support practice around the facilitation of PADs in order to make them more intensive. Introduced in the 1990s in North American mental healthcare services, peer support practitioners have experienced mental health challenges and are trained to support others [50]. They play a central role in promotion of recovery and recovery-oriented practices [51]. These PADs have been tested and improved in Marseille by users, peer-workers and clinicians, leading to a final version used in this study protocol.

To our knowledge, no RCT on PADs has ever been carried out in France.

In order to rigorously evaluate this intervention, a multicentre, randomized controlled study was designed.

## Methods

### Aim

The primary objective is to assess the impact of Psychiatric Advances Directives (PAD) in comparison with routine care by a psychiatrist (Usual Care - UC) on the rate of compulsory admissions to psychiatric hospital over a 12-month follow-up period.

The secondary objectives are: (1) to assess the impact of PAD on care-related outcomes (number of inpatient days, therapeutic alliance), patient reported outcomes (quality of life, satisfaction), mental-health outcomes (recovery, empowerment, awareness of disorders, symptomatology, severity); (2) to measure the cost-effectiveness and cost-utility of PAD in comparison with UC; and (3) to describe changes in professional culture and practices in the different stakeholders, including users, usual professionals and facilitators.

### Design and setting

A multicentre, open-label, randomized, controlled, parallel trial is used to evaluate the effectiveness of PADs. In parallel, a qualitative study is conducted to document the recovery trajectories of individuals in the program, institutional dynamics, and professional practices (including facilitator practice) around PADs.

**Fig. 1:**
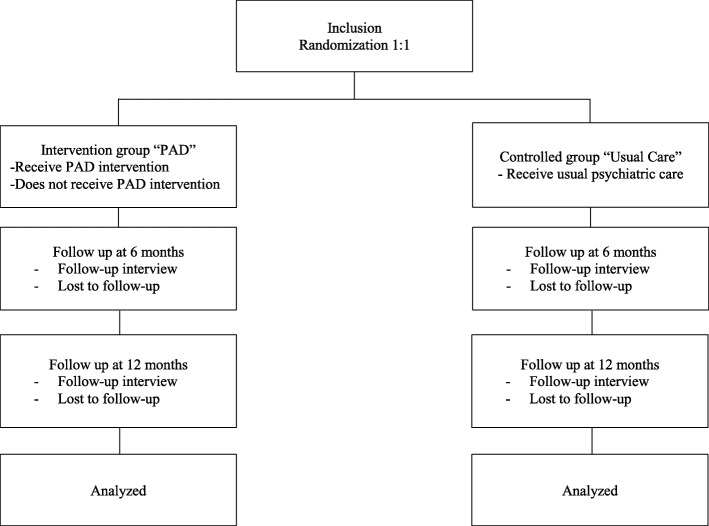
Flow Chart of DAiP Study Design

Subjects are referred by their psychiatrists, recruitment is performed in seven hospitals from three main cities across France: three Public Institution of Mental Health in Marseille (AP-HM, Edouard Toulouse, Valvert), two in Lyon (Le Vinatier, Saint Jean de Dieu ARHM) and two in Paris (GHU and Argenteuil). Trained research assistants check eligibility criteria, describe the trial, answer any questions the candidates may have and obtain their written informed consent. Participants are then randomly assigned to either Psychiatric Advance Directives (Experimental group) or Usual Care (Control group) – see Fig. 1. The 1:1 randomization is stratified per centre and a computer-generated randomization list is created using a permuted block-design. The statistician generated the allocation sequence, psychiatrists and research assistants enrol participants, and research assistants assign participants to the intervention groups according to the randomization list.

**Fig. 2:**
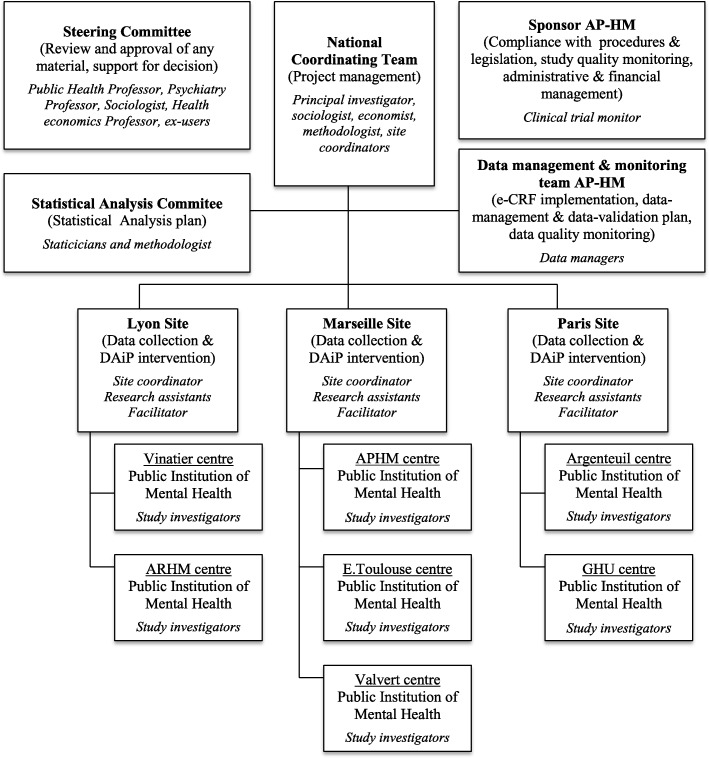
DAiP Study Organizational Structure

The organization of the study and its various committees are represented in Fig. 2.

### Inclusion and exclusion criteria

Inclusion criteria are as follows: being over 18 years old; having a diagnosis of schizophrenia or bipolar I disorder or schizoaffective disorders according to Diagnostic and Statistical Manual of Mental Disorders, fifth edition (DSM-5) criteria; having been involuntarily admitted to hospital within the past 12 months; having decision-making capacity, assessed by a psychiatrist according to the four key components of a capacity evaluation: understanding, appreciation, reasoning, and choice, from the MacArthur Competence Assessment Tool for Clinical Research (MacCAT-CR) [52]; being covered by French government health insurance; and speaking French. Exclusion criteria include the following: being considered unable to provide informed consent and being under guardianship.

### Intervention groups

- Experimental Group: Psychiatric Advance Directives (PAD). Each person assigned to the experimental group is invited to fill in a document describing his or her crisis plan and his or her will or preferences in case of being unable to consent,^1^ and to meet a facilitator specially trained to help them with this. He or she is invited to share the document with his or her psychiatrist and the reliable person he or she has nominated (a healthcare agent). Meetings concerning the document between the peer support worker and the participant take place as soon as the latter decides to do so, and the support offered lasts as long as necessary. Peer support workers are recruited specifically for this study, and trained together for this protocol, on the one hand to ensure both compliance with the protocol and consistency of practice, and on the other hand to better define facilitator practice (what time is needed, what are the obstacles and enablers). Regular exchanges are organised between them, and with the entire research team, via web conferences, and regular meetings during the study.

- Control Group: Usual Care (UC). People assigned to the control group are followed as usual by their psychiatrist.

No concomitant care or interventions are prohibited during the trial participation.

### Participant time line

**Fig. 3:**
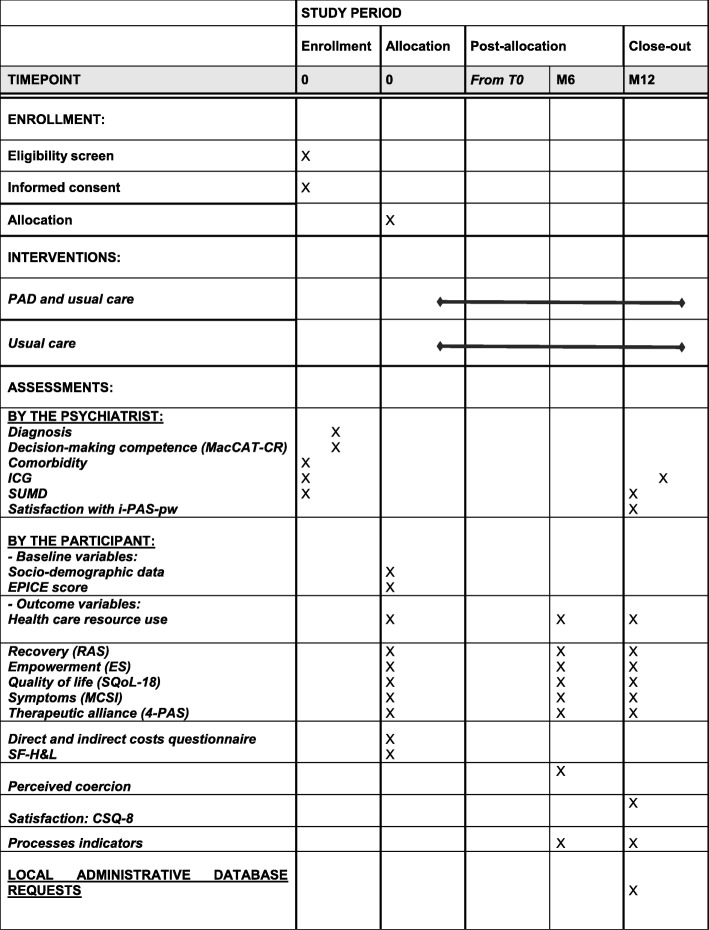
Schedule of enrolment, interventions and assessment. SPIRIT diagram of DAiP Study

Face-to-face interviews to collect quantitative data are planned with research assistants: inclusion and baseline interview (M0), 6-month interview (M6) and 12-month interview (M12) (see Fig. 3).

In the experimental group, research assistants invite participants to meet the facilitator as soon as possible, by proposing an immediate phone call to arrange an appointment. All contact information is provided.

### Outcome measures

#### Primary outcome

The primary outcome is the rate of involuntary admissions to psychiatric hospital at 12 months of follow-up, from local medical databases, crosschecked and completed with declarative data, in particular for admissions in other hospitals (non-local).

#### Secondary outcomes

- Care-related outcomes:
  - ◦ Number of inpatient days measured both from local administrative databases and self-reported data
  - ◦ Perceived coercion, in particular upon compulsory hospitalization
  - ◦ Therapeutic alliance assessed using the 4-Point ordinal Alliance Scale (4-PAS) [53]. This self-rating scale includes two dimensions (empathy experienced and psychoeducation) and a global score. Higher scores indicate higher therapeutic alliance.
  - ◦ Somatic and addictive comorbidity assessed at baseline by the psychiatrist.
- Patient-reported outcomes:
  - ◦ Quality of life assessed using the Schizophrenia Quality-of-Life scale (S-QoL 18 scale), which comprises 18 items evaluating eight dimensions: psychological well-being, self esteem, family relationships, relationships with friends, resilience, physical well-being, autonomy, and sentimental life [54]. Dimension and index scores range from 0, indicating the lowest quality of life, to 100, the highest quality of life. S-QoL-18 has been validated in bipolar disorders [55].
  - ◦ Satisfaction of program users evaluated using the CSQ-8, which is the most frequently used questionnaire in mental health services [56]. The range is from 8 to 32, with higher scores indicating higher satisfaction. Satisfaction of caregivers involved (psychiatrists) is also assessed using an adaptation of the CSQ-8.
- Mental-health outcomes:
  - ◦ Recovery assessed using the Recovery Assessment Scale (RAS), which measures various aspects of recovery from the consumer’s point of view, with a particular emphasis on hope and self-determination [57, 58]. This self-administered instrument comprises 24 items, exploring five domains: personal confidence and hope, willingness to ask for help, goal and success orientation, reliance on others, and not being dominated by symptoms. A higher score indicates better recovery.
  - ◦ Mental health symptomatology assessed using the self-report Modified Colorado Symptom Index (MCSI) [59]. This 14-item tool evaluates how often in the past month an individual has experienced a variety of mental health symptoms, including loneliness, depression, anxiety, and paranoia. Higher scores indicate a greater likelihood of mental health problems.
  - ◦ Empowerment assessed using the Empowerment Scale (ES) [60] which is a specific tool for mental health. It comprises five dimensions: self-esteem, optimism, power, activism, and legitimate anger. Higher scores correspond to higher empowerment.
  - ◦ Awareness of disorders assessed by the psychiatrist at M0 and M12, using an abbreviated version of the Scale to Assess Unawareness of Mental Disorder (SUMD), with nine items describing three dimensions: awareness of the disease, consequences and need for treatment, awareness of positive symptoms, awareness of negative symptoms [61, 62].
  - ◦ Overall severity assessed using Clinical Global Impression (CGI) [63], where the psychiatrist rates on a scale from 1 (healthy, not ill) to 7 (severely ill) at M0 and M12.
- Social outcomes:
  - ◦ Gender, age, education level, nationality, social benefits, wages, employment status, and housing conditions assessed using ad hoc questionnaires.
  - ◦ Deprivation assessed at baseline using the EPICES score (Evaluation of Deprivation and Inequalities in Health Examination Centres). This composite index is commonly used to measure the social and material dimensions of deprivation [64]. The 11-item version has been validated on a large cohort [65]. EPICES score is related to all causes of death, most of the specific causes of death and to premature and avoidable deaths. The higher the score, the more deprived the person is. Authors defined a cutoff value of 30.7 for the threshold defining deprivation.
- Costs:
  - ◦ Direct and indirect costs are measured during the 12-month follow-up period. Direct costs cover the costs related to medical/health services, including days in hospital, emergency department visits, and outpatient visits, and indirect costs mainly related to loss of productivity. Several sources of data will be used: local medical databases (Department of medical information from each centre); medical records; structured interviews with participants, as well as the standardized Short Form-Health and Labor questionnaire (SF-H&L) [66]. The SF-H&L measures productivity losses caused by health problems in general: absenteeism from paid work, production losses without absenteeism from paid work and hindrance in the performance of paid and unpaid work.
  - ◦ Cost-utility analysis, measured in quality-adjusted life years (QALYs), using the EQ5D scale that applies utility values to each state of health [67].

### Process measurements

Evaluation of the allocation in PAD group (i.e. whether or not participants have completed the PAD document, shared them and with whom, met with the facilitator, and the time spent with the facilitator) is also recorded in both groups at 6 and 12 months. In addition, the sociologist will compare the dynamics between the centres for the different levels of implementation identified by Nicaise: completion, content, access and honoring [37].

### Sample size

Sample size was calculated to detect a reduction of 30% in the rate of compulsory admissions to psychiatric hospital during the follow-up period of 12 months between the two groups (consistent with similar RCTs: 29, 39, 40), with a reference point of 42.6% [68]. To obtain a significance level of 2.5% and power of 80% with equal allocation to two arms, each arm of the trial would require 182 people. To allow for a potential 10% of people being lost to follow-up, 200 will be recruited per arm, i.e. 400 in total. Considering the inclusion potential of each participating centre, inclusion is planned to last for a 12-month period. The expected period of participation for each included individual is 12 months.

To promote participant retention, research assistants were recruited for their interpersonal skills. In addition, participants will receive €20 for each interview. For people lost to follow-up, the primary outcome will be collected from local medical databases.

### Statistical analysis

The data will be summarized using the mean, median, standard deviation and range for quantitative data, and frequencies for categorical data. Analysis of the primary and secondary criteria will be performed on the intent-to-treat population. In addition, complementary per protocol analyses will be performed.

Comparisons between the two groups for each outcome will be performed using Student’s t-tests or Mann-Whitney-Wilcoxon tests for quantitative variables, and chi-squared or Fisher’s exact tests for proportions. Non-parametric tests will be used for data that is not normally distributed. Multivariate analyses will be performed primarily using negative binomial or Poisson regression models (for overdispersed data) for the number of involuntary admissions and a gamma distribution for the number of inpatient days, adjusting for the lengths of follow-up. Explanatory variables will be selected among those for which the *P*-value is below or equal to 0.20 in univariate analysis. The results will be presented in the form of standardized beta coefficients.

We will use a GEE approach with an exchangeable correlation matrix, which assumes that patient outcomes from the same centre are correlated but are independent from patient outcomes in different centres.

Statistical significance is defined as *P* < 0.05. Statistical analyses will be performed using SPSS Statistics for Windows, Version 20.0 (SPSS Inc. Chicago, IL, USA) or STATA 16 (StataCorp. 2019. Stata Statistical Software: Release 16. College Station, TX: StataCorp) LLC.

### Cost utility analysis

Health benefits and healthcare costs related to interventions targeted to prevent persons with severe mental illness from involuntary hospital admissions should be analysed in order to inform decision makers, psychiatrists, judges, and relatives of their efficiency [67]. Balancing patient safety with health-related quality of life, hospital length-of-stay (LOS) and associated costs is vital [69]. The aim of the economic analysis is therefore to investigate whether intensive Psychiatric Advance Directives with the peer support would result in significant improved health-related quality of life and healthcare savings compared to usual care.

To quantify the efficiency of PAD from society’s point of view, we propose to calculate the incremental cost-utility ratio (ICUR) of the intervention compared to Usual Care. Incremental benefits will be measured in quality-adjusted life years (QALYs), which is particularly relevant here since an expected reduction in involuntary admission rate associated with enhanced autonomy and self-determination would have rapid positive impacts on the patient’s QoL. The time horizon will be 12 months after randomization.

QALYs is calculated by multiplying the number of years of life gained by a health-state associated utility value (corresponding to patient’s quality of life) during the period under consideration. Preference-based utility scores will be calculated using the three-level version of the EuroQol five-dimensional questionnaire (EQ-5D-3 L). This is a validated questionnaire that assesses a participant’s health status in terms of 5 dimensions (mobility, self-care, routine occupations, pain and discomfort, and anxiety and depression) [70].

All healthcare resources will be observed, quantified and value added throughout the period between baseline and the end of the RCT follow-up. These healthcare costs will include resources for PAD intervention (including professional time and training), in-patient resources (including compulsory and non-compulsory hospital admissions), outpatient and home care, and pharmacy claims over the 12-month follow-up period. Cost resources will be valued using data from the French National Health system and hospital databases, databases for medical and paramedical acts, French National official list prices for drugs and registers of pharmaceutical specialities. Indirect costs will be estimated using the SF-H&L questionnaire but not value added as recommended by the French Health Authority [71].

Sensitivity analysis will be carried out to test the robustness of the results. One-way sensitivity analysis and tornado diagrams will be used to identify thresholds for factors influencing the ICUR. Probabilistic sensitivity analysis, using the non-parametric bootstrap method, will be carried out to generate mean expected ICURs and to determine whether uncertainty or variation in the data affect ICURs [72]. In addition, cost-effectiveness acceptability curves will be drawn to represent decision uncertainty surrounding cost-effectiveness estimates [73].

### Qualitative analysis

The qualitative evaluation of the PAD program is carried out by a sociologist for three groups of stakeholders: care users, health professionals and institutions. It aims to understand, from a recovery perspective, the effects of the program on these different actors. Focus groups and semi-structured interviews with intervention group participants will be done at each site to identify the organizational and institutional dynamics that are specific to each centre. There will also be individual interviews with psychiatrists, care coordinators, peer support workers and participants. At least 28 care users (4 per centre) will be interviewed twice using the life story method to describe how the PAD program fits into their trajectory. This evaluation process is combined with a participatory approach. The various stakeholders are invited to participate in the reflection on the implementation of the program and its generalization. Inductive thematic analysis, including constant comparison methods [73], will be used to analyse data specifically related to explaining the trial outcomes.

### Data management

The data entry is done through an e-CRF (electronic Case Report Form) developed using the open source web application REDCap. The access to this application is secure and is done with a user id and a password. Each user, and its role in the study, is clearly defined. Data captured through the software is backed up daily on a secure network. The database of the study is stored on a specific directory of the server, administered by the Digital Services Direction of the sponsor. Data quality is detailed in data-management and data-validation plans, approved by the principal investigator of the study. Data managers structure the data of the included-patients, and check their coherence and reliability. The data-manager provides the database to the biostatistician once it is cleaned and frozen.

The data management and monitoring team will oversee the intra-study data sharing process. Principal Investigator will be given access to the cleaned data sets. To ensure confidentiality, data dispersed to project team members will be blinded of any identifying participant information.

### Monitoring

In the event of interim analysis or definitive discontinuation of the study, the National coordinating team will obtain competent authorities the authorisation. As this research is part of the research involving the human “at risk and minimal constraints” (categorie 2 in Jardé Law), and in accordance with the regulations in force, it will be up to the investigator to declare any serious adverse event that occurred during the investigations according to the internal procedure of declaration of a serious adverse event associated with the care of his institution.

The sponsor will conduct study quality monitoring. The project can be audited by the French competent authority in case of non-respect of the safety or the rights of the participant.

### Dissemination policy

The national coordinating team and the steering committee will review all presentations and publications before their publication. All presentations and publications are expected to protect the integrity of the major objective of the study.

The study results will be released to the participating psychiatrists, professionals, participants, relevant policymakers, and the community through users’ associations.

## Discussion

Advance directives are popular among care users [74], and seem promising in guiding the mental healthcare system towards more shared decision-making and more active participation of care users. We expect that our study will bring some discussions in France around these ideas. The role of facilitator has been highlighted to ensure the intensity of the PADs, which leads to greater effectiveness in reducing hospital admissions. Article 12 of the United Nations (UN) Convention on the Rights of Persons with Disabilities (CRPD) clearly highlights the risk of “undue influence” and the need of protection in supported decision-making, to fully respect rights, will and preferences [75]. Indeed, coercion in psychiatry includes compulsion, but also pressure, persuasion, interpersonal leverage, inducement and threats (“if you refuse the medication, you will go back to hospital”) [76]. Classical healthcare professionals or family can easily influence choices, more or less significantly, and more or less voluntarily [77, 78]. By employing peer support workers with own life experience to support the drafting of PADs, we assume that there is less risk of putting pressure on the person during this exercise with someone who has personally suffered from coercion and has personally experienced the importance of personal choice and responsibility in recovery [79], as is emphasised in literature on support with decision-making [80].

Potential barriers to PADs include the physician’s attitude, lack of time of professionals, lack of people’s engagement, lack of access to the document during a crisis or follow-up, inappropriate requests, lack of communication between carers, or potential conflict of interest between the physician and the person [81–84]. The recruitment of a trained facilitator dedicated to this mission could mediate and overcome a number of these obstacles. However this may be not sufficient because shared decision-making is described as a process of enabling clients to participate actively and meaningfully in their treatment. This requires accessible information, choices and integration of care, and therefore the full cooperation of healthcare professionals. In our study, participating psychiatrists are voluntary and theoretically aware, but are not required to undergo any significant training or comply with the document. The preliminary implementation in Marseille enrolled volunteers from a peer support group, followed by recovery-oriented psychiatrists, and the transfer of this towards a usual setting could be more challenging than expected.

A sensitive question in this study is linked to the evaluation of mental capacity, because psychosis and involuntary admission were showed to be the strongest risk factors for decision-making incapacity [1]. However, literature shows that mental capacity can be reliably assessed, even if no gold standard exists as yet [85]. In this DAiP study, evaluation of competence by a psychiatrist with the help of MacCAT-CR is an inclusion criterion. Beyond the baseline assessment, this issue of decision-making competence is of particular interest and will be investigated in semi-structured interviews and focus groups. Indeed, other moral dilemmas may arise in cases where a person rejects support and/or intimates a wish to place himself or herself in a situation of danger, exploitation, abuse or undue influence [86].

The experimental intervention is defined as being a “complex intervention” [37] i.e. an intervention that involves a number of separate but interacting components that are likely to be important to the success of the intervention. If RCTs have been described as being a reliable method to evaluate complex interventions [87], their evaluation raises methodological questions [88]. In our study, we address this challenge by collecting data from qualitative and quantitative approaches.

A qualitative approach is also particularly interesting to supplement the quantitative approach because, as mentioned above, constraint is highly influenced by context. The combination of methods and their triangulation throughout the research process ensures a more detailed understanding of the conditions under which PADs can be deployed in a non-binding legislative context.

Furthermore, this type of research will assess the potential for generalisation of PADs from a public policy point of view. Indeed, the choice of experimental design was guided by establishing the highest level of evidence in order to influence policy decisions (evidence-based policy making), but RCTs have many blind spots that can be enlightened by a qualitative approach, e.g. transformation of practices, facilitators and obstacles perceived by the stakeholders.

The question arises of whether such research is necessary since this type of intervention does not basically require scientific evidence to be implemented. Moreover, it is already public policy in several countries, such as in England and Wales (Mental Health Act) or in India (Indian Mental Healthcare Act) without being evidence-based. This highlights the position of this topic at the crossroads of human rights, science and justice.

Strengths of our research protocol include firstly the number of participating centres and the number of psychiatrists involved (at least 40) from different backgrounds, reflecting the diversity of practices in France; secondly the complementarity of quantitative and qualitative approaches with strong collaboration between researchers; thirdly the involvement of care users at several levels of the study: conception of the document, construction of the protocol, and peer support work to help participants from the experimental group.

Limits of the study include firstly the lack of blinding, which only concerns the primary outcome for participants; secondly the implementation in hospitals in large cities, which may not be representative of smaller hospitals in smaller cities; thirdly the restriction to 3 types of diagnosis, which do, however, represent half of the inpatient psychiatric treatment in France [13].

To conclude, reducing coercion in mental healthcare requires urgent mobilization. Psychiatric Advance Directives drafted with support of a peer-worker could contribute to the necessary cultural change that is needed to enhance autonomy and self-determination, as international (UN CRPD) and local human rights defenders strongly advocate.

Research on interventions to reduce constraint is highly contextual, influenced by different service configurations, different mental health laws, different social policies, and culture [75]. More research on PADs in different contexts and cultures like the present trial is therefore needed.

## Supplementary information

**Additional file 1.** Directives Anticipées incitatives en Psychiatrie (DAiP). Document of Psychiatric Advance Directives used in DAiP Study. Four pages in French.

**Additional file 2.** Notice d’information destinée à la personne participant à l’étude. Description: Informed consent form used in DAiP Study. Seven pages in French.

## Data Availability

Materials are available upon reasonable request from the author.

## Abbreviation

4-PAS: 4-Point ordinal Alliance Scale
BP1: Bipolar I Disorder
CSQ-8: Consumer Satisfaction Questionnaire-8
DSM V: Diagnostic and Statistical Manual of Mental Disorders V^th^ edition
EPICES: Evaluation of Deprivation and Inequalities in Health Examination Centres
EQ-5D-3 L: EuroQol Five-Dimensional Three-Level version
ES: Empowerment Scale
ICER: Incremental Cost-Effectiveness Ratio
ICG: Clinical Global Impression
ICUR: Incremental Cost-Utility Ratio
JCP: Joint Crisis Plan
LOS: Hospital Length-Of-Stay
MacCAT-CR: MacArthur Competence Assessment Tool for Clinical Research
MCSI: Modified Colorado Symptom Index
PAD: Psychiatric Advance Directive
QALY: Quality-Adjusted Life Years
QoL: Quality Of Life
RAS: Recovery Assessment Scale
RCT: Randomised Controlled Trial
SCZ: Schizophrenia
SCZaff: Schizoaffective Disorders
SF-H&L: Short Form-Health and Labor questionnaire
S-QoL 18: Schizophrenia Quality-of-Life scale – 18 items
SUMD: Scale to Assess Unawareness in Mental Disorder
UC: Usual Care
UN-CRPD: United Nation - Convention on the Rights of Persons with Disabilities

## Footnotes

1 The document figures in Additional file 1 (in French). It contains the name and contact of the healthcare agent –the participant’s “reliable person”, the name of the people to be contacted or not in case of a crisis, the triggering signs and what helps or does not help at this stage, what helps in case of a crisis and what does not, the procedure to be followed in the event of danger, the treatments and others things accepted during a crisis and those to be avoided, and the choice of patient care facilities. Objections, values, and principles can also be stipulated in the document.
