# Supplementary material for: Psychiatric advance directives for people living with schizophrenia, bipolar I disorders, or schizoaffective disorders: Study protocol for a randomized controlled trial – DAiP study
Source: BMC Psychiatry. 2019 Dec 27;19:422. doi: 10.1186/s12888-019-2416-9 (PMC6935101; doi:10.1186/s12888-019-2416-9)
Supplement: Supplementary file 1 — Additional file 1. Directives Anticipées incitatives en Psychiatrie (DAiP). Document of Psychiatric Advance Directives used in DAiP Study. Four pages in French. [file 12888_2019_2416_MOESM1_ESM.pdf]

Directives anticipées incitatives en Psychiatrie

Personne concernée et personne(s) de confiance

Je soussigné(e), ………………………………………………………………………………………
né(e) le ………../………../………………… à ……………………………………………………
désigne comme personnes de confiance, en application de l'article L. 311-5-1 du code de l'action sociale et des familles :

	**Nom**	**Contact (n° de mobile)**	**Nature du lien**	**Rôle**
**Personne de confiance n°1**				
**Personne de confiance n°2**				
**Personne à prévenir n°1**				
**Personne à prévenir n°2**				

Ajouter le cas échéant d'autres personnes de confiance/à prévenir sur papier libre, signé, joint au présent document.

Afin de faire valoir les directives anticipées ci-rédigées, avec mon consentement lors de sa rédaction. Etant entendu que les directives anticipées incitatives en psychiatrie n'ont pas de valeur contraignante pour les proches et les soignants.

Fait le……../………/…………… à…………………………………… seul(e) ou avec/en présence de (Nom, prénom des témoins)…………………………………………………………………………

Nom(s) et signatures des personnes de confiance :

- ……………………………………………………………………………………………………
- ……………………………………………………………………………………………………
- ……………………………………………………………………………………………………
- ……………………………………………………………………………………………………

IMPORTANT : Je suis capable de décider sauf quand (présentez les situations, « états », signes indiquant que vous devez laisser quelqu'un décider pour vous/que vous préférez « passer le relais à une personne de confiance) **:**
……………………………………………………………………………………………………
……………………………………………………………………………………………………
……………………………………………………………………………………………………
……………………………………………………………………………………………………
……………………………………………………………………………………………………
……………………………………………………………………………………………………
……………………………………………………………………………………………………
……………………………………………………………………………………………………
……………………………………………………………………………………………………
……………………………………………………………………………………………………
……………………………………………………………………………………………………

Signes avant-coureurs amenant à la crise (à remplir)

1 –

3 –

2 –

4 –

Nouveaux signes (« mise à jour ») :

Ce qui m'aide (à mettre en lien avec les signes avant-coureurs) :

1 –

3 –

2 –

4 –

Ce qui ne m'aide pas (à mettre en lien avec les signes avant-coureurs) :

1 –

3 –

2 –

4 –

Signaux de fin de crise, quand ça va mieux

1 –

3 –

2 –

4 –

En cas de mise en danger (auto ou hétéro agressivité)

A faire :

-
-
-

A ne pas faire :

-
-
-

En cas de crise :

Ce qui m'aide :	Ce qui ne m'aide pas :

Traitements ou soins qui m'aident/ne m'aident pas

Les traitements qui m'aident, qui peuvent être utilisés

(Relatif à article 1111.4 du Code de la Santé Publique sur le choix du traitement)

Traitement/Soin	Posologie	Usage/effet	Remarque

Les traitements qui ne m'aident pas, qui ne doivent pas être utilisés

Traitement/soin	Posologie	Usage/effet	Remarque

Je souhaite être conduit dans un lieu de soin : **OUI** □ **NON** □

Lieux de soin (hôpitaux, cliniques, services, etc.) où je veux être pris(e) en soin :

-
-
-
-
-
-

Remarques :……………………………………………………………………………………………………..

……

……

……

Lieux où je refuse d'être pris(e) en soin :

-
-
-
-
-
-

Remarques :……………………………………………………………………………………………………
……
……
……

Si je refuse tout lieu de soin :

Détailler la solution envisagée : où je préfère aller ; Avec qui ? Ce qu'il faut faire, etc.

……
……
……
……
……
……
……
……
……

Par qui je souhaite/par qui je refuse d'être accompagné

Personnel médical

(relatif à l'article 6,R.4127-6 du code de la santé publique sur le choix du médecin)

Par qui je veux être accompagné(e) :

-
-
-

Par qui je refuse d'être accompagné(e) :

-
-
-

Personnel paramédical (infirmiers etc.), médiateurs, travailleurs sociaux, etc. :

Par qui je veux être accompagné(e) :

-
-
-

Par qui je refuse d'être accompagné(e) :

-
-
-

Autres remarques personnelles, choses à savoir, notes…

……
……
……
……
……
……
……
……
……
……
