# Supplementary material for: Psychiatric advance directives for people living with schizophrenia, bipolar I disorders, or schizoaffective disorders: Study protocol for a randomized controlled trial – DAiP study
Source: BMC Psychiatry. 2019 Dec 27;19:422. doi: 10.1186/s12888-019-2416-9 (PMC6935101; doi:10.1186/s12888-019-2416-9)
Supplement: Supplementary file 2 — Additional file 2. Notice d’information destinée à la personne participant à l’étude. Description: Informed consent form used in DAiP Study. Seven pages in French. [file 12888_2019_2416_MOESM2_ESM.pdf]

Notice d'information destinée à la personne participant à l'étude

Etude interventionnelle multicentrique, randomisée, comparative, prospective évaluant l'impact sur le parcours de soin d'un programme de rédaction accompagnées de directives anticipées incitatives psychiatriques pour les personnes souffrant de schizophrénie, de troubles bipolaires ou de troubles schizoaffectifs.

Promoteur :
Assistance Publique des Hôpitaux de Marseille, 80 Rue Brochier, 13354 MARSEILLE Cedex 5

Investigateur coordonnateur : Dr Aurélie TINLAND
Pôle psychiatrique universitaire Solaris, hôpital Sainte-Marguerite, APHM, 261 Bd Sainte Marguerite, 13009 Marseille.
EA 3279 : CEReSS - Centre d'Etude et de Recherche sur les Services de Santé et la Qualité de vie, Aix-Marseille Université, Faculté de Médecine - Secteur Timone, 27 bd Jean Moulin, 13005 Marseille

Madame, Monsieur,

Nous vous proposons de lire attentivement cette notice d'information qui a pour but de répondre aux questions que vous seriez susceptibles de vous poser avant de prendre votre décision de participation à l'étude « Directives Anticipées incitatives en Psychiatrie - DAiP ».
Vous pourrez à tout moment durant l'étude de vous adresser au Dr/Pr ____________ pour lui poser toutes les questions supplémentaires.
Votre participation est entièrement volontaire. Si vous ne désirez pas prendre part à cette étude, vous continuerez à bénéficier de la meilleure prise en charge médicale possible, conformément aux connaissances actuelles.

<u>En quoi consiste cette étude ?</u>
La schizophrénie, les troubles bipolaires ou les troubles schizoaffectifs entraînent des périodes de stabilité et des périodes de crise. Ces alternances varient beaucoup selon les individus : certaines personnes ne connaissent qu'une crise dans leur vie, d'autres en traversent plusieurs par an. Elles entraînent parfois des moments où les capacités de décision sont atteintes, pouvant entraîner des hospitalisations sous contraintes.
Les grandes études épidémiologiques et de nombreux témoignages montrent que les personnes atteintes de ces troubles peuvent se rétablir, vivre une vie satisfaisante, et ce d'autant plus qu'elles sont pleinement actrices de leurs vie.
<u>L'objectif de l'étude</u> est d'observer si le fait de rédiger pendant une période de stabilité des « directives anticipées incitatives psychiatriques » change le parcours de soin des personnes qui les rédigent.
Pour cela, deux groupes de personnes sont constituées : celles qui sont incitées à rédiger leurs directives anticipées, et celles qui ne sont pas incitées à le faire. Les données des deux groupes seront comparées à la fin de l'étude.
Nous souhaitons impliquer 400 personnes dans cette étude, 200 dans 2 groupes, à partir de 3 villes : Lyon, Marseille, et Paris.

<u>Qu'implique la participation à cette étude ?</u>
Si vous acceptez de participer à cette étude, vous devrez **signer le consentement éclairé** pour confirmer que vous avez bien été informé du projet de recherche et que vous ne vous opposez pas au recueil de vos données et à leur utilisation dans le cadre de cette recherche.

Si vous acceptez de participer à cette étude, vous devrez **rencontrer un enquêteur tous les 6 mois : au début de l'étude, 6 mois après, et 12 mois après.**
Celui-ci vous rencontrera avec une grille de questions sur votre parcours de soin et votre ressenti, votre vécu (notamment avec des échelles de qualité de vie, de rétablissement, ou encore de satisfaction).
L'enquêteur sera attentif à vos souhaits, et les respectera. Vous pourrez par exemple remplir seul certaines de ces grilles, si vous le souhaitez. Vous serez bien sûr libre de ne pas répondre aux questions auxquelles vous ne souhaitez pas répondre.
Toutes vos réponses seront anonymisées (plus de détails dans le paragraphe « droits » p2).
L'entretien ne durera pas plus d'une heure.
Vous bénéficerez d'un remboursement de vos frais de transports occasionnés par votre venue dans le centre pour la réalisation des visites, sur présentation des justificatifs.
Si vous êtes volontaire, vous pourrez aussi être sollicité pour des entretiens individuels plus poussés, appelés « semi-structurés », qui seront enregistrés par le chercheur, ou encore pour des entretiens réalisés en groupe, appelés « focus group » ; ainsi que pour une démarche dite de « recherche action participative », où vous participerez à l'élaboration de la recherche.

Il y a deux groupes dans l'étude. Vous saurez à l'issue de cet entretien dans quel groupe vous serez assigné, selon une liste pré-établie, inconnue par les personnes qui vous présentent l'étude.

- **Si vous êtes inclus dans le groupe « directives anticipées incitatives en psychiatrie » :** Vous recevrez un document à remplir. L'assistance d'un médiateur de santé vous sera proposée. Il connaît très bien le document, et la manière de le remplir. Il pourra répondre à toutes vos questions. Vous pourrez rencontrer ce médiateur dans un lieu de votre choix, et prendre avec lui le temps nécessaire à cette rédaction. Vous pourrez partager le document rempli avec votre psychiatre si vous le souhaitez.
- **Si vous êtes inclus dans le groupe « prise en charge habituelle » :** Il n'y aura pas de modification de votre prise en charge habituelle.

La participation à cette étude vous empêche de participer simultanément à une autre étude de recherche.

<u>Quels sont les bénéfices que vous pouvez attendre de votre participation à l'étude ?</u>

Cette étude vous propose de participer à la recherche en psychiatrie.
En répondant aux questions, vous nous aiderez à faire progresser la connaissance sur la vie des personnes qui vivent avec des troubles psychiques, sur le respect de leurs droits, et sur leurs parcours de soin.
Les entretiens individuels, en groupe ou la participation à la « recherche action participative » sont des espaces d'expression en présence de sociologues formés, et ces espaces peuvent être très intéressants pour ceux qui souhaitent s'en saisir.
Pour les personnes du groupe « directives anticipées incitatives en psychiatrie » : vous aurez accès à un dispositif innovant, que vous nous aiderez à mieux comprendre, et vous pourrez être accompagné dans cet exercice par un médiateur. Vous aurez l'occasion de réfléchir et de partager avec votre entourage et les soignants des informations permettant une meilleure gestion de vos troubles et une prise en charge plus conforme à ce que vous souhaitez.
Pour les deux groupes, vous pourrez vous exprimer face à des enquêteurs formés à l'écoute et au soutien.

<u>Quels sont les risques que vous pouvez craindre, liés à votre participation à l'étude ?</u>

Pour les personnes du groupe « prise en charge habituelle », les risques sont faibles. Vous pourrez ressentir une déception de ne pas accéder au dispositif expérimental, même si vous pouvez rédiger vos directives anticipées incitatives psychiatriques par vous-même. Il n'y a aucun changement dans votre parcours de soin.
Pour les personnes du groupe « directives anticipées incitatives en psychiatrie » : vous pourrez être déçu par le dispositif, le médiateur de santé, qui est un professionnel atypique, ou encore le fait que

vos directives ne soient pas respectées par les soignants ou l'entourage. De plus, le fait de rédiger le document nécessite de se remémorer des épisodes douloureux de sa vie, comme les hospitalisations sous contrainte.
Pour les deux groupes, il y a des contraintes, liées au fait de devoir se rendre aux rendez-vous prévus avec l'enquêteur.

Que se passe-t-il si vous décidez de ne pas participer à l'étude ?
La participation à l'étude est volontaire. Vous êtes libre de refuser ou d'interrompre votre participation à cette étude à tout moment, sans encourir aucune responsabilité ni aucun préjudice de ce fait, et sans avoir à vous justifier. Cela n'altèrera pas la qualité des soins qui vous seront prodigués et ne modifiera pas vos relations avec l'ensemble de l'équipe soignante.

Quels sont vos droits en tant que participant(e) à cette recherche ?

L'investigateur coordonnateur de cette étude est le Dr Aurélie TINLAND.
Le promoteur de cet essai est l'Assistance Publique-Hôpitaux de Marseille.
Si vous le souhaitez, vous pourrez être informé des résultats de l'étude.

Les résultats de cette recherche vont être obtenus grâce aux réponses que vous aurez données à l'enquêteur. Ces réponses sont des données personnelles.

Les données enregistrées à l'occasion de cette étude feront l'objet d'un traitement informatisé dont le responsable est le promoteur de cette recherche (Assistance Publique – Hôpitaux de Marseille)

Concernant le traitement informatisé, Il sera réalisé conformément à la méthodologie de référence homologuée par la Commission nationale de l'informatique et des libertés et établie en concertation avec le comité consultatif sur le traitement de l'information en matière de recherche dans le domaine de la santé, élaborée dans le but de simplifier les formalités (Délibération n°2016-262 du 21 juillet 2016 portant modification de la méthodologie de référence pour les traitements de données personnelles opérés dans le cadre des recherches biomédicales. Méthodologie de référence MR-001).

Il sera également réalisé conformément au règlement général sur la protection des données (RGPD) n° 2016/679 entré en vigueur le 25 mai 2018. Ce traitement des données repose sur l'exécution d'une mission d'intérêt public dont est investi le responsable du traitement (article 6.1.e du RGPD), dans le domaine de la santé publique et à des fins de recherche scientifique (alinéas i et j de l'article 9.2 du RGPD).

Conformément aux dispositions de la loi relative à l'informatique, aux fichiers et aux libertés (Loi n°78-17 du 6 janvier 1978 modifiée par la loi n° 2004-801 du 6 août 2004), et à l'article 13 du règlement général sur la protection des données n°2016/679 entré en vigueur le 25 mai 2018, vous disposez d'un droit d'accès, de rectification et d'effacement des données personnelles collectées ainsi que d'un droit de limitation et d'opposition au traitement de ces données. Vous disposez également d'un droit d'opposition à la transmission de ces données, couvertes par le secret professionnel, susceptibles d'être utilisées dans le cadre de cette recherche et d'être traitées. En cas de difficulté en lien avec la gestion des données collectées, vous pouvez adresser une réclamation auprès du délégué à la protection des données au sein de l'Assistance Publique – Hôpitaux de Marseille (Pr J. Sarles), par mail à dpd@ap-hm.fr ou auprès de la CNIL.

La durée de conservation des données collectés est de 15 ans conformément à la réglementation en vigueur.

Pour participer à cette étude vous devez être affilié à un régime de sécurité sociale.

Cette recherche relève de l'application du Code de la Santé Publique. Il est soumis au nouveau dispositif réglementaire qui s'applique aux recherches « impliquant la personne humaine», à savoir La loi n° 2012-300 du 5 mars 2012 relative aux recherches impliquant la personne humaine (dite loi Jardé) telle que modifiée par l'ordonnance n° 2016-800 du 16 juin 2016, et ses décrets d'application.
Ces informations sont consultables sur le site Internet de Legifrance (www.legifrance.gouv.fr)

Cette étude a reçu un avis favorable du comité de protection des personnes Sud-Ouest et Outre-mer 4 le 31/08/2018. Selon la réglementation en vigueur, l'Agence Nationale de Sécurité du Médicaments et des produits de santé a été informé de cette étude le 11/09/2018. Le promoteur, représenté par l'Assistance Publique des Hôpitaux de Marseille, adresse 80 rue Brochier 13005 Marseille. En tant que promoteur, il a souscrit un contrat d'assurance auprès de SHAM (N° de contrat 156.923).

Vous pouvez également accéder directement ou par l'intermédiaire d'un médecin de votre choix à l'ensemble de vos données médicales (article L 1111-7 du Code de la Santé Publique).

En cas de dommage vous pouvez avoir accès à une indemnisation selon les modalités des articles L. 1121-10 et L.1142-3 du Code de la Santé Publique.

Ce document vous appartient et nous vous invitons à en discuter avec votre médecin et/ou avec vos proches.
La participation à cette étude nécessite la signature du formulaire ci-après nommé « Formulaire de recueil du consentement »

En cas d'interruption de l'étude, les informations vous concernant seront conservées sauf opposition de votre part. Vous serez libre d'utiliser le document que vous avez rempli.

Dans l'éventualité d'un arrêt des financements du fait d'un nombre insuffisant de participants, l'étude pourrait être suspendue par le promoteur. Dans ce cas, les visites de suivi prévues avec l'enquêteur pourraient être annulées. Par contre, les directives anticipées ayant été rédigées et partagées ne seraient pas annulées. Elles sont un outil de prévention de la crise pour la personne, son entourage ainsi que pour son psychiatre, et peuvent être utilisées de la même manière hors du cadre de l'étude.

Toute information nouvelle survenant pendant votre participation et pouvant modifier la décision de votre participation vous sera donnée

A tout moment avant et en cours d'étude vous avez la possibilité de poser des questions concernant la recherche, vos données et vos résultats auprès de :

Pour obtenir d'autres informations
Pour demander l'accès à vos données
Pour disposer des résultats globaux de l'étude
Dr Tinland laboratoire de santé publique EA3279, boulevard Jean Moulin, 13005 Marseille
Secteur Timone, 27 bd Jean Moulin, 13005 Marseille
Pôle psychiatrique universitaire Solaris, hôpital Sainte-Marguerite, APHM, 261 Bd Sainte Marguerite, 13009 Marseille
Téléphone : 04.91.32.45.60 / 04.91.74.67.31

Formulaire de recueil de consentement pour la personne participant à l'étude

Etude interventionnelle multicentrique, randomisée, comparative, prospective évaluant l'impact sur le parcours de soin d'un programme de rédaction accompagnées de directives anticipées psychiatriques pour les personnes souffrant de schizophrénie, de troubles bipolaires ou de troubles schizoaffectifs.

Promoteur :
Assistance Publique des Hôpitaux de Marseille, 80 Rue Brochier, 13354 MARSEILLE Cedex 5
Investigateur coordonnateur : Dr Aurélie TINLAND
Pôle psychiatrique universitaire Solaris, hôpital Sainte-Marguerite, APHM, 261 Bd Sainte Marguerite, 13009 Marseille.
EA 3279 : CEReSS - Centre d'Etude et de Recherche sur les Services de Santé et la Qualité de vie, Aix-Marseille Université, Faculté de Médecine - Secteur Timone, 27 bd Jean Moulin, 13005 Marseille

Je soussigné(e) :
Mme/Mr, ___________________________________ (*nom, prénom*), né(e) le : ____________
déclare accepter participer à la recherche impliquant la personne humaine interventionnelle de catégorie 2 intitulée « Directives Anticipées incitatives en Psychiatrie – DAiP » organisée par le Docteur Aurélie TINLAND, dans les conditions précisées ci-dessous ;

1. Avoir compris que je bénéficie d'un délai de réflexion entre le moment où l'information m'a été donnée et le moment de la signature de ce document,

2. Avoir été informé que j'ai la possibilité de retirer mon accord de participation à tout moment, sans justification aucune et sans que cela ne modifie mes relations avec le personnel soignant ni ma prise en charge,

3. Avoir été informé que je conserve tous mes droits garantis par la loi (La loi n° 2012-300 du 5 mars 2012 relative aux recherches impliquant la personne humaine (dite loi Jardé) / Code de la Santé Publique ; titre II du livre premier relatif aux recherches impliquant la personne humaine),

4. Avoir été informé que cette recherche a reçu un avis favorable du Comité de Protection des personnes Sud-Ouest et Outre-mer 4 le 31/08/2018 et a été déclarée à l'Agence Nationale de Sécurité du Médicaments et des produits de santé le 11/09/2018,

5. Avoir été informé de l'objectif, du déroulement, des avantages et des inconvénients relatifs à cette recherche, et avoir été informée qu'elle se déroulera selon les Bonnes Pratiques Cliniques définies dans le Bulletin Officiel publié par le Ministère des Affaires Sociales et de l'Emploi,

6. Avoir pu poser toutes les questions que je voulais et avoir reçu des réponses adaptées que j'ai clairement comprises, et avoir noté que je pouvais compléter ces renseignements tout au long de l'étude auprès du Pr/Dr ……………..,

7. Avoir été informé que le Promoteur de cette étude est représenté par l'Assistance Publique des Hôpitaux de Marseille (DRCI 80, rue Brochier, 13354 Marseille Cedex 05), a souscrit, à ce

titre, un contrat d'assurance "Responsabilité Civile" conformément à la loi en vigueur auprès de la SHAM (contrat n°156.923),

8. Avoir été informé de l'utilisation anonyme des données me concernant, recueillies dans le cadre de cette recherche par traitement informatisé. La présentation des résultats de l'étude ne pourra permettre mon identification directe ou indirecte,

9. Avoir été informé que ces données ne pourront être consultables que par les investigateurs de l'étude et le promoteur ou par des personnes mandatées par le promoteur et astreintes au secret professionnel, ou des personnes mandatées par les autorités administratives, sanitaires et judiciaires,

10. Avoir été informé que je pourrais si je le souhaite accéder à ces données, les vérifier et demander des modifications si nécessaire, conformément à la loi en vigueur (garantis par les articles 39 et 40 de la loi n°78-17 du 6 janvier 1978 relative à l'informatique, aux fichiers et aux libertés et des lois subséquentes dont la loi n°2004-801 du 6 août 2004 et à l'article 13 du règlement général sur la protection des données n°2016/679 entré en vigueur le 25 mai 2018),

11. Avoir noté que toute nouvelle information survenant en cours d'étude, susceptible notamment de remettre en cause ma participation, me sera communiquée dès que possible,

12. Avoir compris que le promoteur ou l'investigateur peuvent décider à tout moment d'interrompre l'étude,

13. Avoir compris que mon consentement ne dégage pas le promoteur et les investigateurs de leurs responsabilités,

14. Etre affilié à un régime de sécurité sociale ;

15. Avoir été informé que les résultats globaux de l'étude pourront m'être communiqués conformément à l'article L1122-1 du Code de Santé Publique ;

16. Avoir compris qu'il m'était possible de joindre le Pr/Dr…………… pour toute information complémentaire au numéro suivant …………………

17. Avoir compris que si j'accepte de participer à cette recherche, je dois signer ce document et que mon représentant légal doit également signer si je suis sous curatelle,

18. Ne pas permettre mon identification ;

19. Ayant disposé d'un temps de réflexion suffisant avant de prendre ma décision,

J'accepte librement et volontairement de participer à la recherche DAiP dans les conditions précisées ci-dessus

Fait à ____________________ **Le** _________________

Nom et signature du patient ______________________

Nom et signature du médecin investigateur __________________________________

- **<u>Uniquement dans le cas où le patient est sous curatelle</u>**

Le consentement est donné par l'intéressé assisté de son curateur informé de la participation du patient sous curatelle.

Fait à ____________________ **Le** _______________

Nom du participant ______________________

Nom et signature du médecin investigateur __________________________________

Nom du curateur ______________________

Fait en deux exemplaires originaux (un conservé par le médecin investigateur et un conservé par le patient).
